# Transcriptomic analysis reveals the potential biological mechanism of AIS and lung adenocarcinoma

**DOI:** 10.3389/fneur.2023.1119160

**Published:** 2023-05-17

**Authors:** Rong-Xing Qin, Yue Yang, Jia-Feng Chen, Li-Juan Huang, Wei Xu, Qing-Chun Qin, Xiao-Jun Liang, Xin-Yu Lai, Xiao-Ying Huang, Min-Shan Xie, Li Chen

**Affiliations:** ^1^Department of Neurology, The First Affiliated Hospital, Guangxi Medical University, Nanning, Guangxi Zhuang Autonomous Region, China; ^2^Collaborative Innovation Centre of Regenerative Medicine and Medical BioResource Development and Application Co-constructed by the Province and Ministry, Guangxi Medical University, Nanning, Guangxi Zhuang Autonomous Region, China; ^3^Guangxi Key Laboratory of Regenerative Medicine and Guangxi Collaborative Innovation Center for Biomedicine, Guangxi Medical University, Nanning, Guangxi Zhuang Autonomous Region, China

**Keywords:** lung adenocarcinoma, acute ischemic stroke, transcriptome, cellular senescence, bioinformatics

## Abstract

**Introduction:**

Acute ischemic stroke (AIS) and lung adenocarcinoma (LUAD) are associated with some of the highest morbidity and mortality rates worldwide. Despite reports on their strong correlation, the causal relationship is not fully understood. The study aimed to identify and annotate the biological functions of hub genes with clinical diagnostic efficacy in AIS and LUAD.

**Methods:**

Transcriptome and single-cell datasets were obtained from the Gene Expression Omnibus (GEO) and The Cancer Genome Atlas (TCGA). We identified the differentially expressed genes (DEGs) upregulated in AIS and LUAD and found 372 genes intersecting both datasets. Hub genes were identified using protein-protein interaction (PPI) networks, and the diagnostic and prognostic utility of these hub genes was then investigated using receiver operating characteristic (ROC) curves, survival analysis, and univariable Cox proportional hazard regression. Single-cell analysis was used to detect whether the hub genes were expressed in tumor epithelial cells. The immune microenvironment of AIS and LUAD was assessed using the CIBERSORT algorithm. The protein expression of these hub genes was tracked using the Human Protein Atlas (HPA). We calculated the number of positive cells using the digital pathology software QuPath. Finally, we performed molecular docking after using the Enrichr database to predict possible medicines.

**Results:**

We identified the molecular mechanisms underlying hub genes in AIS and LUAD and found that *CCNA2, CCNB1, CDKN2A*, and *CDK1* were highly expressed in AIS and LUAD tissue samples compared to controls. The hub genes were mainly involved in the following pathways: the cell cycle, cellular senescence, and the HIF-1 signaling pathway. Using immunohistochemical slices from the HPA database, we confirmed that these hub genes have a high diagnostic capability for AIS and LUAD. Further, their high expression is associated with poor prognosis. Finally, curcumin was tested as a potential medication using molecular docking modeling.

**Discussion:**

Our findings suggest that the hub genes we found in this study contribute to the development and progression of AIS and LUAD by altering the cellular senescence pathway. Thus, they may be promising markers for diagnosis and prognosis.

## 1. Introduction

Human health is in peril from conditions like cancer and stroke (1). Over 795,000 acute ischemic stroke (AIS) occurrences are reported each year in the United States alone, making it the second most frequent cause of mortality and long-term disability worldwide (2). A study found that in 2020, the incidence and mortality rates of stroke in China were 505.2 and 343.4 per 100,000 person-years, respectively (3). The proportions of acute ischemic stroke due to atherosclerosis and cardiogenic embolism were 39% and 22%, respectively (4). AIS is a multifactorial disease influenced by atherosclerosis, hypertension, alcohol consumption, smoking, and D-dimer levels. In many low-income and middle-income nations, stroke incidence and fatality rates have increased in recent decades (5). Genetic variables, which are essential for determining the etiology of AIS, are suggested by epidemiological research to be connected to the prevalence of stroke (6).

One of the molecular mechanisms of AIS includes immune activation, which can lead to both neuroprotective and neurotoxic effects (7, 8). Thrombolysis and antiplatelet therapy are currently the main treatment methods for stroke, but it is unclear whether these treatments improve prognosis in patients (9). Lung cancer is the leading cause of cancer-related deaths globally. Additionally, lung cancer has a terrible prognosis (10, 11). The most common histological subtype of malignant lung cancer is lung adenocarcinoma (LUAD). Deaths are mostly attributed to cancer cell invasion and distant metastasis (12). Gene abnormalities are the initial factors that influence the biological behavioral changes of heterogenic cancer cells, including cell signaling and the immune microenvironment. According to reports, several genes play a role in the development of LUAD tumors (13–15). During tumor progression, changes in genes that mediate important biological processes can occur, resulting in the rapid proliferation of malignant cells. However, the mechanism by which this process occurs is still unclear (16). As a result of studies on the molecular basis of lung cancer, programmed death ligand 1 and epidermal growth factor receptor tyrosine kinase inhibitors are two newly emerging treatment targets. Even though lung cancer treatment choices have increased thanks to genetic testing, novel targeted therapy, and immunotherapy, its 5-year survival rate is as low as 20% in many nations (10, 17), and the recurrence rate remains high (18). Recently, an increasing number of reports have documented AIS caused by malignant tumors (19) and demonstrated that patients with lung cancer and metastatic cancer display a higher risk of stroke. Notably, the cumulative incidence of stroke among lung cancer patients was 5.1% (20). The connection between lung cancer and stroke significantly decreased the patients' life longevity and quality (19). LUAD may lead to multiple acute cerebral infarction lesions involving multiple arterial blood supply areas (21). *CCNB2* may be a possible target against lung cancer and AIS, according to earlier research (22). Moreover, the stroke is the second most frequent neurological condition associated with death in cancer patients. Risk factors for cancer-related AIS include high levels of D-dimer, fibrinogen breakdown products, and C-reactive protein (23). LUAD and AIS are closely associated with the occurrence of gene abnormalities. However, the regulatory mechanisms of the genes involved have not yet been elucidated. Therefore, it is crucial to comprehend how they interact and find relevant indicators to provide a plan for diagnosis and treatment. In this study, we used bioinformatics to identify hub genes in AIS and LUAD, investigated their biological functions, and determined their clinical significance.

## 2. Materials and methods

### 2.1. Data sources

Using the expression data of AIS in the GEO database, we discovered the expression data and clinical details about LUAD on the Xena platform. The inclusion criteria were as follows: (i) case and control groups, (ii) microarray data of mRNA, (iii) the specimen was a peripheral blood sample or tissue, and (iv) *Homo sapiens*. The GSE122709 dataset was included according to the above criteria, including five control peripheral blood samples and 10 AIS peripheral blood samples. The Cancer Genome Atlas (TCGA)-LUAD array from the Xena platform was selected, which included 59 surrounding normal samples and 524 tumor samples. Furthermore, we also downloaded LUAD validation datasets GSE42127, GSE68465, GSE50081, GSE13231, and GSE31210. In addition, we downloaded the validation datasets of AIS (GSE58294 and GSE140275) from GEO. All of these datasets met the above inclusion criteria. Datasets obtained were subjected to logarithmic-scale conversion and other data processing. We downloaded the single-cell dataset GSE146100 and then did quality assurance and data filtering.

### 2.2. Differential gene expression analysis

We performed a difference analysis using the R package “limma”. Differential genes were defined as those with a *P*-value < 0.05 and |log2FC (fold change)| ≥ 1 values. The number of 372 differentially expressed genes (DEGs) was found after using a Venn diagram to find the intersecting genes.

### 2.3. Functional analysis

We examined the Gene Ontology (GO) and Kyoto Encyclopedia of Genes and Genomes (KEGG) of the elevated DEGs using the R package “clusterProfiler.” *P*-values < 0.05 were used to determine whether an enrichment function was significant. Cellular components, molecular functions, and biological processes are all included in the GO category. The top 10 GO biological processes were chosen in ascending order of *P*-values. Ten significant KEGG in total were found. The increased DEGs were annotated using the disease ontology (DO) analysis R package “DOSE”. Gene set enrichment analysis (GSEA) was used to define hub genes to pinpoint the signaling pathways connected to AIS and LUAD. Patients were split into two groups (control and case group) based on the levels of expression of the four hub genes. GSEA analysis reveals differences in signaling pathways. The gene sets with a significant enrichment were then sorted. GSEA was used to investigate the relationship between illness categories and biological processes. The cutoff was established at *P* < 0.05.

### 2.4. Building a protein-protein interaction (PPI) network and choosing hub genes

We chose DEGs from the KEGG pathway for cellular senescence for the PPI network. Using the STRING internet database (http://stringdb.org), we looked for connections between proteins. We constructed a PPI network for genes with a rating scale >0.4 using Cytoscape.

### 2.5. Immune infiltration analysis

CIBRSORT was used to estimate the proportion of immune cells in the AIS gene expression matrix. We use the R package “ggplot2” to draw the boxplots. Statistics were deemed significant at *P* < 0.05.

### 2.6. Receiver operating characteristic (ROC) analysis

We investigated the mRNA expression levels of the hub genes in patients with AIS and LUAD using ROC curves and boxplots. To create the ROC curves, we utilized the R package “pROC”.

### 2.7. Survival analysis

TCGA-LUAD expression data and clinical data were used to conduct a survival analysis using the R packages “survminer” and “survival”. The prognosis of LUAD patients was examined using Kaplan-Meier (KM) analysis. The time-dependent ROC curve (timeROC) method was applied to assess the hub genes prediction accuracy at 1, 3, and 5 years.

### 2.8. Analysis of single-cell data and identification of cell subpopulations

We downloaded the scRNA-seq dataset GSE146100 of LUAD. We used the TISCH database (http://tisch.comp-genomics.org) and Maestro analysis for quality control, clustering and cell type annotation, making the expression of four hub genes in the epithelial cell type comparable.

### 2.9. Protein expression and validation

Information about the distribution of proteins in human organs and cells can be found in the HPA database (https://www.proteinatlas.org). We acquired immunohistochemistry (IHC) pictures of LUAD tissues and investigated the differential protein expression of hub genes in matched HPA normal tissues. QuPath, a program for digital pathology, was utilized to count the positive cells. We analyzed The Clinical Proteomic Tumor Analysis Consortium (CPTAC) dataset using the UALCAN tool (https://ualcan.path.uab.edu). The protein levels of the four hub genes were compared between LUAD and normal tissues.

### 2.10. Drug prediction and molecular docking

We used the DSigDB dataset in Enrichr (http://amp.pharm.mssm.edu/Enrichr) for drug prediction of hub genes. Structural information of curcumin was downloaded from the pubChem compound database (https://pubchem.ncbi.nlm.nih.gov). The 3D structures of the proteins of the four hub genes were downloaded using the PDB database (https://www.rcsb.org). “Dockeasy” (https://www.dockeasy.cn/) was used to achieve molecular docking.

### 2.11. Statistical analysis

All data were analyzed using R version 4.2.1. Statistics were deemed significant at *P* < 0.05.

## 3. Results

### 3.1. Differently expressed genes (DEGs) in AIS and LUAD

**Figure 1:**
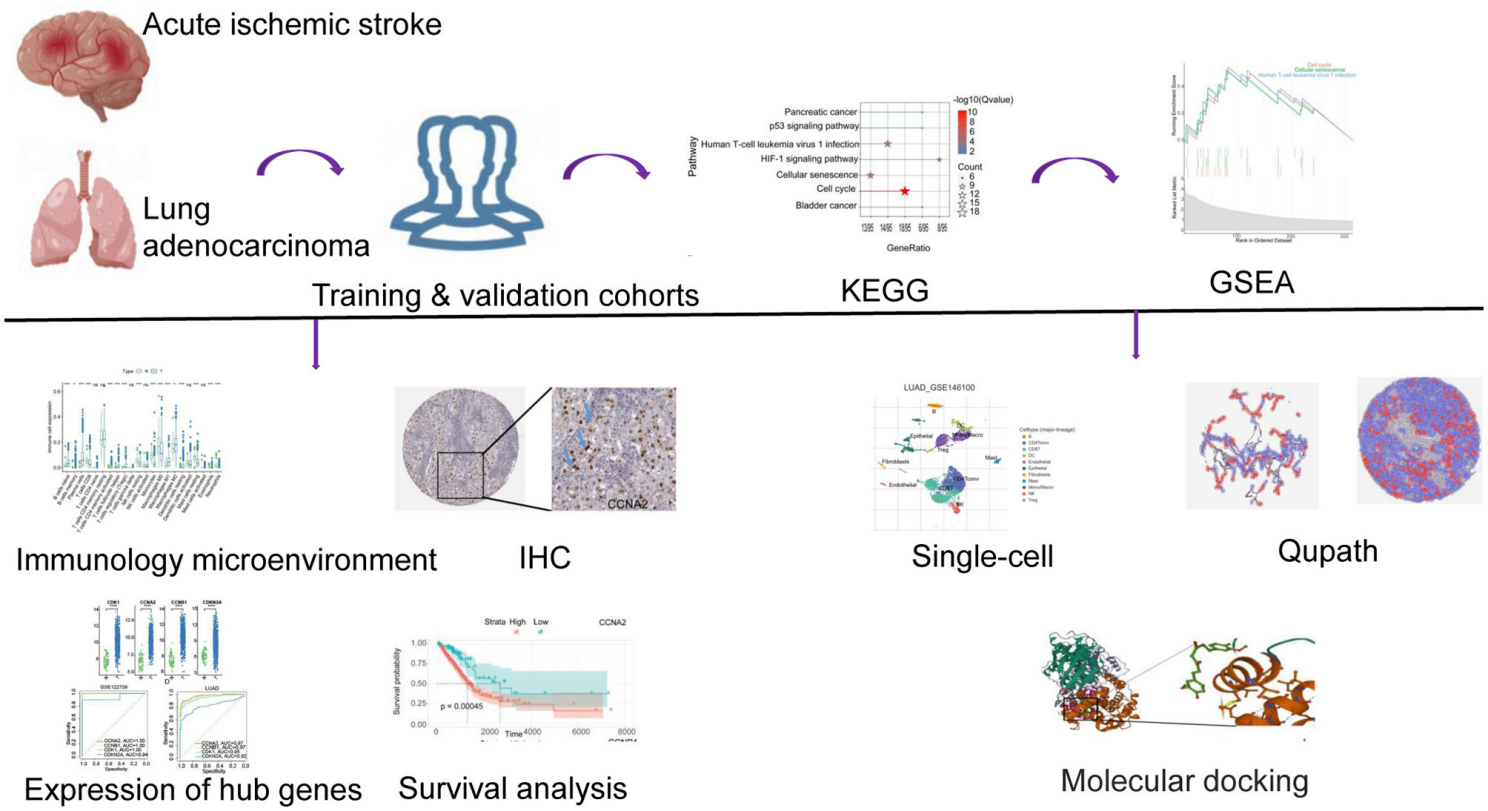
The flow chart for this study.

The flowchart of our study is as follows (Figure 1). The GEO database provided the AIS-related GSE122709 dataset. Expression data for LUAD were obtained from the Xena database. The specific sample information for the two datasets is listed below (Table 1). Using the standard of |log2FC| > 1 and *P* < 0.05, 4452 and 2013 upregulated DEGs were identified in the AIS and TCGA-LUAD datasets, respectively. We found 372 upregulated DEGs intersecting the two datasets, which may be connected to the pathophysiology of AIS and LUAD.

**Table 1:** The sample information of AIS and LUAD.

**Accession**	**Controls**	**Patients**	**Upregulated genes**
GSE122709	5	10	4,452
TCGA-LUAD	59	524	2,013

### 3.2. Functional analyses

**Figure 2:**
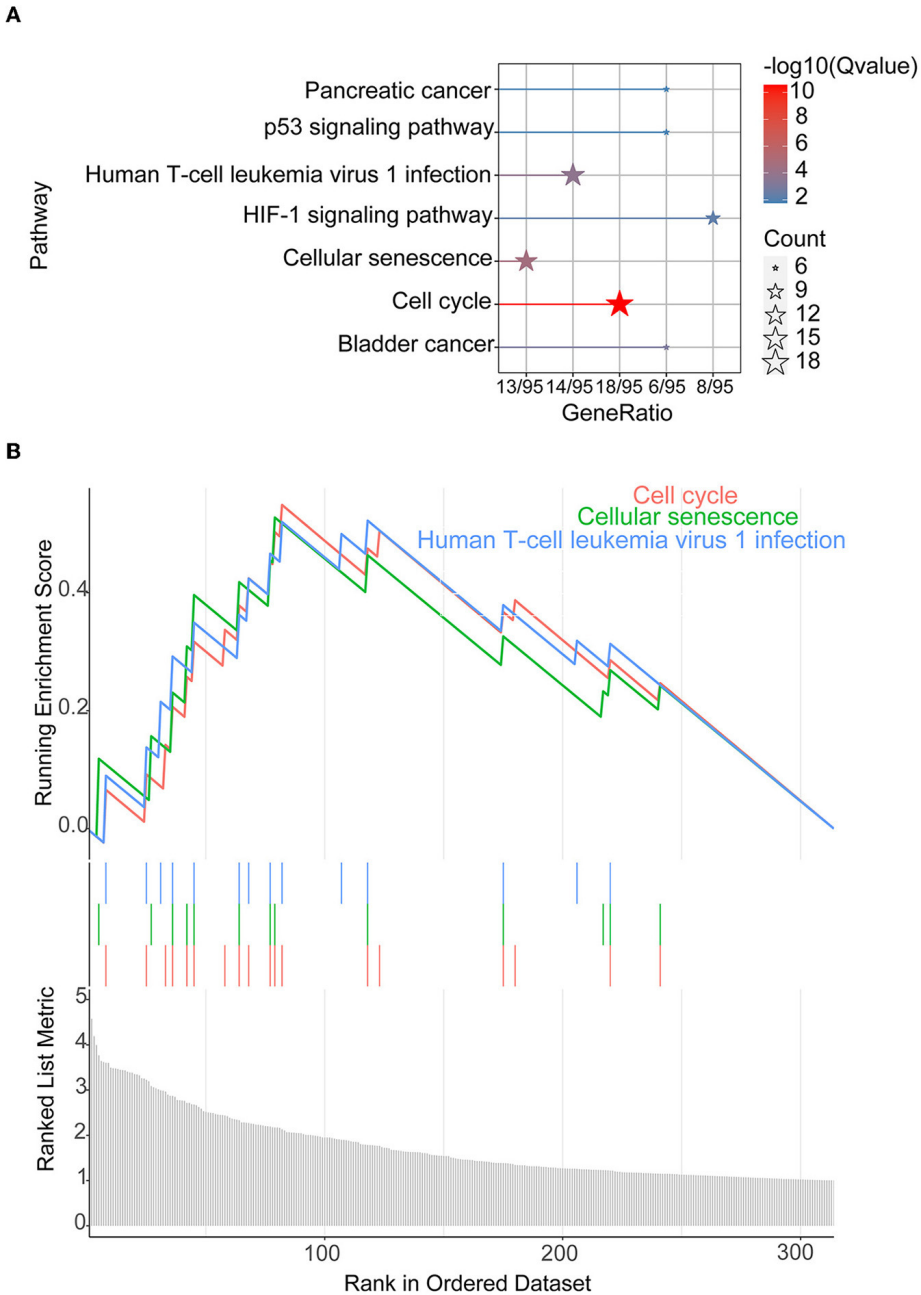
KEGG and GSEA. **(A)** KEGG of 372 upregulated DEGs in AIS and LUAD. **(B)** We conducted GSEA on 372 upregulated DEGs in LUAD.

**Figure 3:**
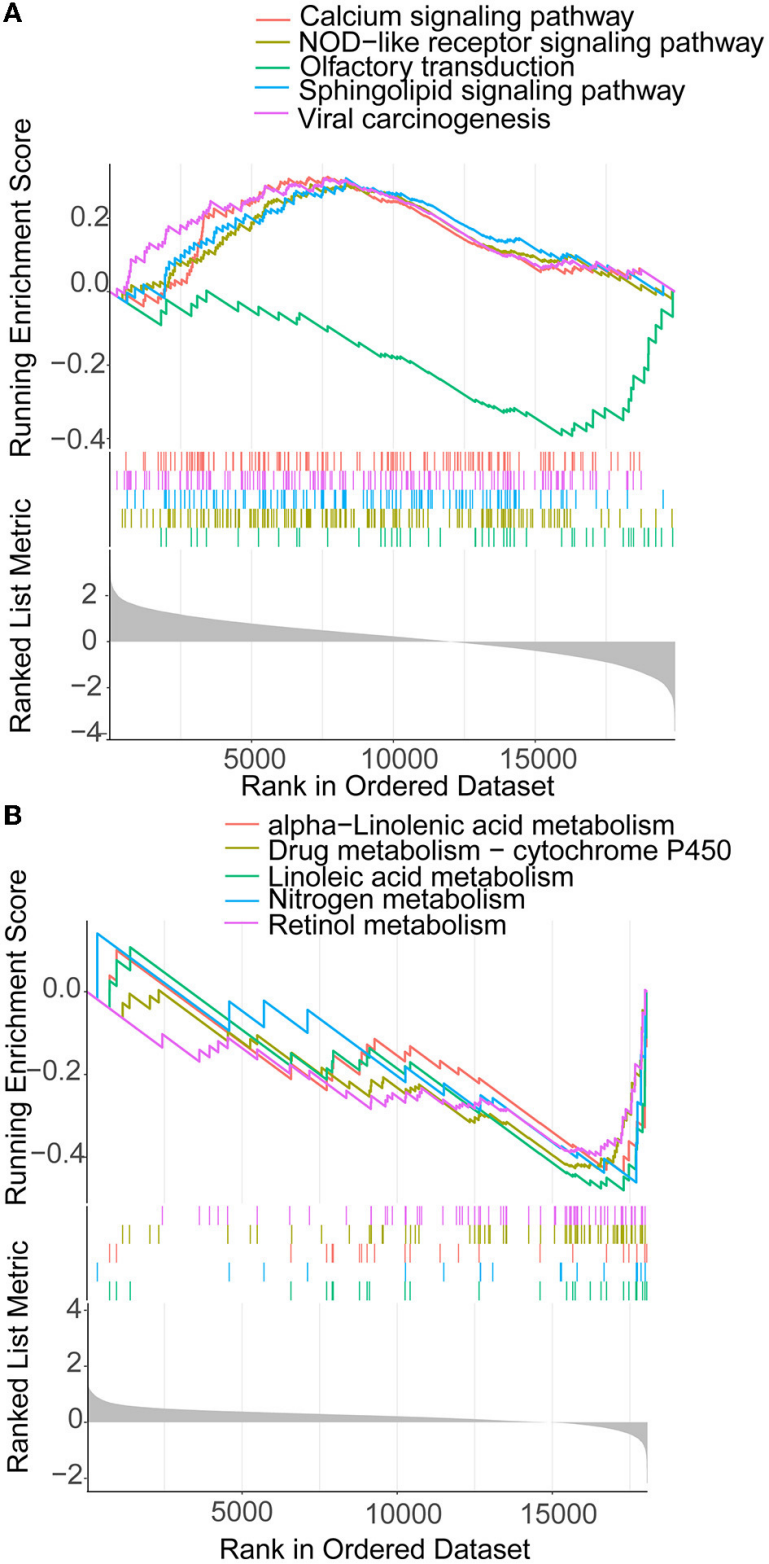
GSEA of *CDKN2A* in AIS and LUAD. **(A)** *CDKN2A* is involved in the main pathway of AIS. **(B)** *CDKN2A* is involved in the main pathway of LUAD.

The 372 elevated DEGs were examined to seek into the shared biological processes and signaling pathways. The GO analysis revealed that these genes were mainly enriched in nuclear, organic fission, and mitotic nuclear divisions. The main KEGG enrichment pathways were the cell cycle, cellular senescence, and the HIF-1 signaling pathway in AIS and LUAD (Figure 2A). DO analysis revealed that the DEGs were linked to many malignancies. These results imply that LUAD and AIS development and incidence may be caused by the differential expression of these genes. These pathways were primarily enriched in the cell cycle, cellular senescence, and human T-cell leukemia virus 1 infection in LUAD, according to the 372 elevated DEGs discovered by GSEA (Figure 2B). In contrast, no related pathways were enriched in the 372 upregulated DEGs in AIS. In AIS, *CDKN2A* is mainly localized in the calcium signaling pathway, NOD-like receptor signaling pathway and olfactory transduction pathway (Figure 3A). In LUAD, *CDKN2A* was mostly implicated in alpha-linolenic acid metabolism, drug metabolism-cytochrome P450, and Linoleic acid metabolism (Figure 3B). We selected DEGs in the cellular senescence pathway using KEGG for analysis. We discovered hub genes and elucidated the potential link between DEG-encoded proteins. Using the STRING database, we built a PPI network and identified the top 10 genes (Table 2), and then we defined four hub genes *CCNA2, CCNB1, CDKN2A*, and *CDK1* from the top 10 genes using Cytoscape.

**Table 2:** The top 10 genes in the PPI network for LUAD and AIS.

**Rank**	**Node**
1	*CCNA2*
1	*CCNB1*
1	*CDKN2A*
1	*CDK1*
5	*CCNE2*
5	*CCNB2*
5	*FOXM1*
5	*E2F1*
9	*MYBL2*
9	*CHEK2*

### 3.3. Validation of hub genes expression and assessment of diagnostic value

**Figure 4:**
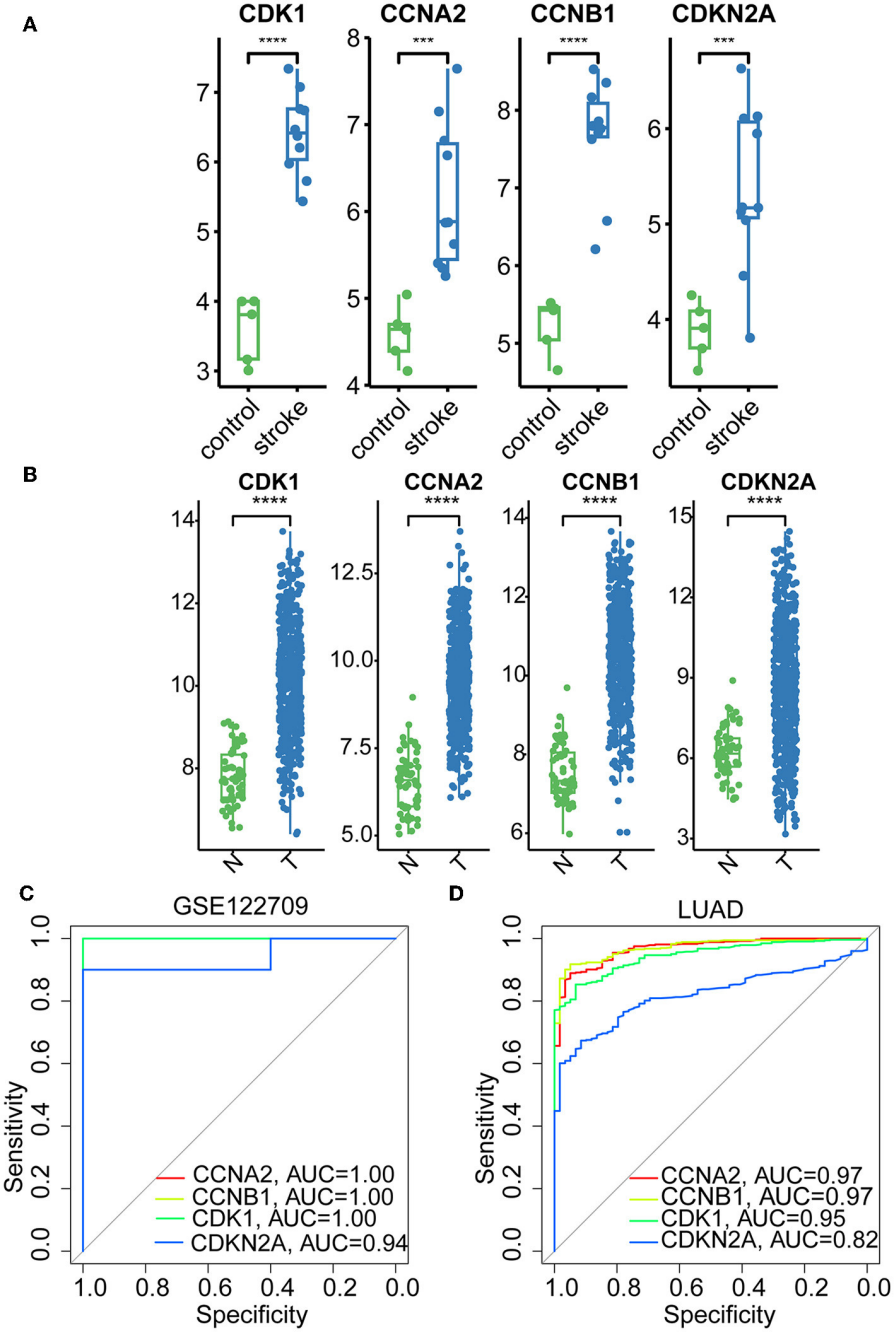
Boxplots and ROC curves of four hub genes in AIS and LUAD. **(A)** Boxplots of the four hub genes in AIS. **(B)** Boxplots of the four hub genes in LUAD. **(C)** ROC curves of the four hub genes in AIS. **(D)** ROC curves of the four hub genes in LUAD (***, *p* < 0.001; ****, *p* < 0.0001; N, normal; T, tumor).

**Figure 5:**
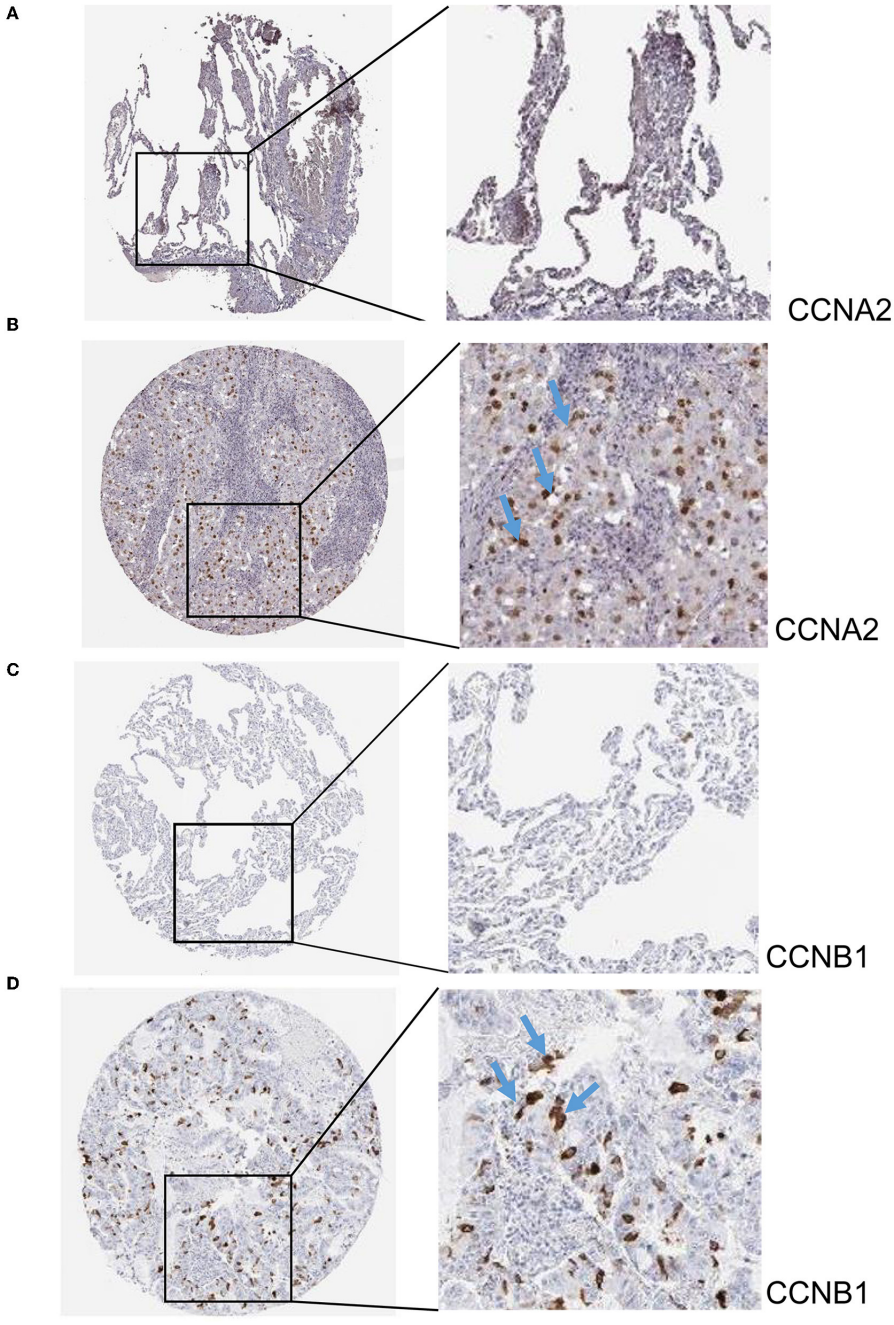
Immunohistochemistry slices of *CCNA2* and *CCNB1*. **(A)** Immunohistochemistry slices of normal lung tissues of *CCNA2*. **(B)** Immunohistochemistry slices of LUAD of *CCNA2*. **(C)** Immunohistochemistry slices of normal lung tissues of *CCNB1*. **(D)** Immunohistochemistry slices of LUAD of *CCNB1*.

**Figure 6:**
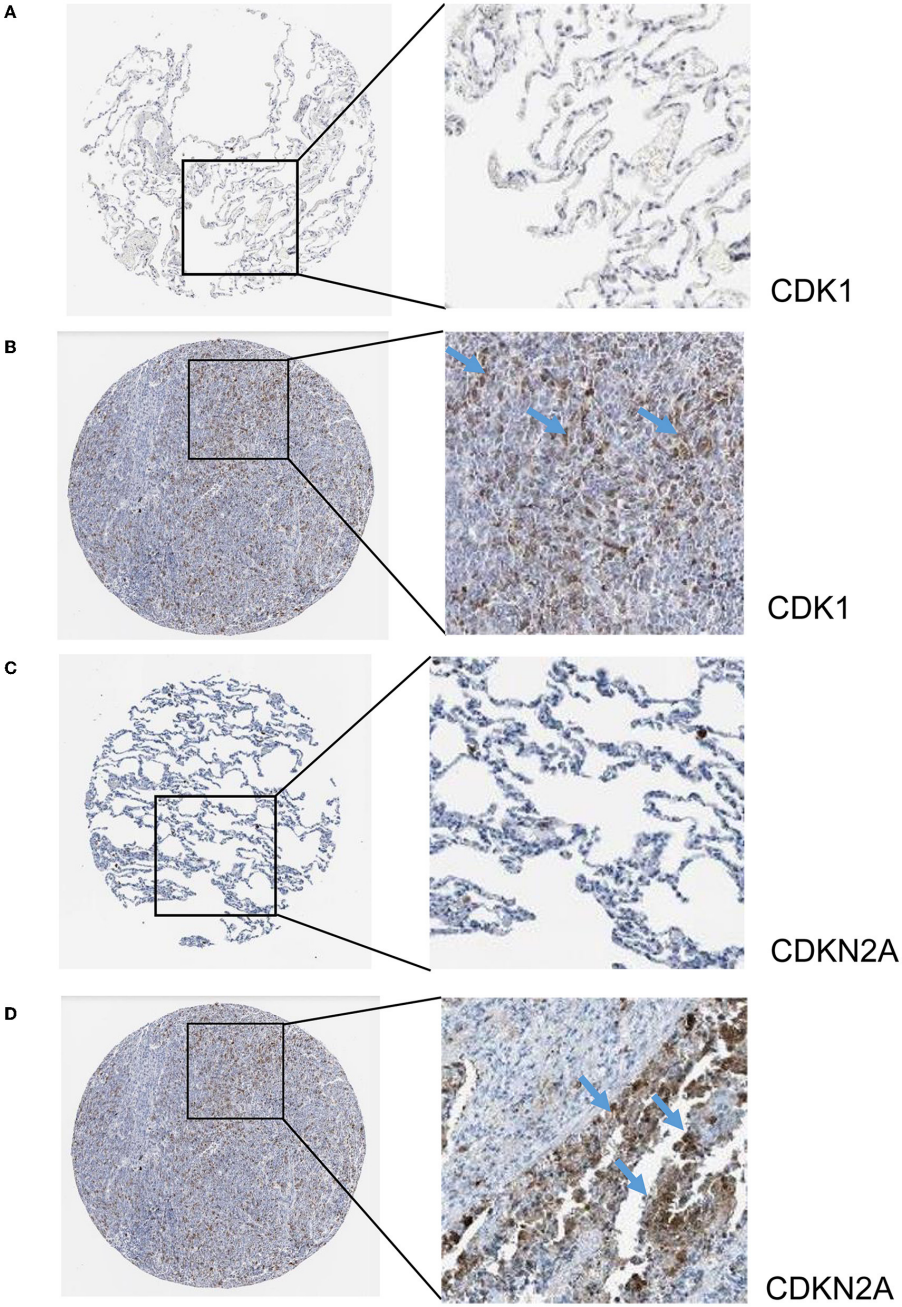
Immunohistochemistry slices of *CDK1* and *CDKN2A*. **(A)** Immunohistochemistry slices of normal lung tissues of *CDK1*. **(B)** Immunohistochemistry slices of LUAD of *CDK1*. **(C)** Immunohistochemistry slices of normal lung tissues of *CDKN2A*. **(D)** Immunohistochemistry slices of LUAD of *CDKN2A*.

The hub genes displayed considerably higher expression in LUAD and AIS patients compared to the control group. In addition, we checked the expression of four hub genes in AIS using the validation datasets (GSE58294 and GSE140275). According to our findings, AIS patients had higher levels of *CCNA2, CCNB1*, and *CDK1* expression compared to the control group (Supplementary Figure 1). However, the four hub genes are insignificant in the GSE140275 dataset, which would account for the limited sample size. Single-cell data analysis revealed that 10996 cells were divided into 11 clusters. The results indicated that *CCNA2, CCNB1, CDK1* and *CDKN2A* were expressed in cancer epithelial cells (Supplementary Figure 2). The diagnostic effectiveness of these hub genes was further investigated using ROC curves, which revealed that these genes have great diagnostic efficacy for LUAD and AIS (Figure 4). This work analyzed immunohistochemistry slices from LUAD patients and typical tissues from healthy people. The four hub genes' protein expression levels were higher in LUAD tissues than those in healthy tissues (Figures 5, 6). We used the digital pathology software QuPath to count the number of positive cells in LUAD and normal lung tissue figures. As a result, we observed that there were more positive cells overall in the LUAD field than there were in the normal lung tissue (Supplementary Figure 3). It's showed that four hub genes had higher protein levels in LUAD compared with normal tissues (Supplementary Figure 4).

### 3.4. Survival analysis and validation

**Figure 7:**
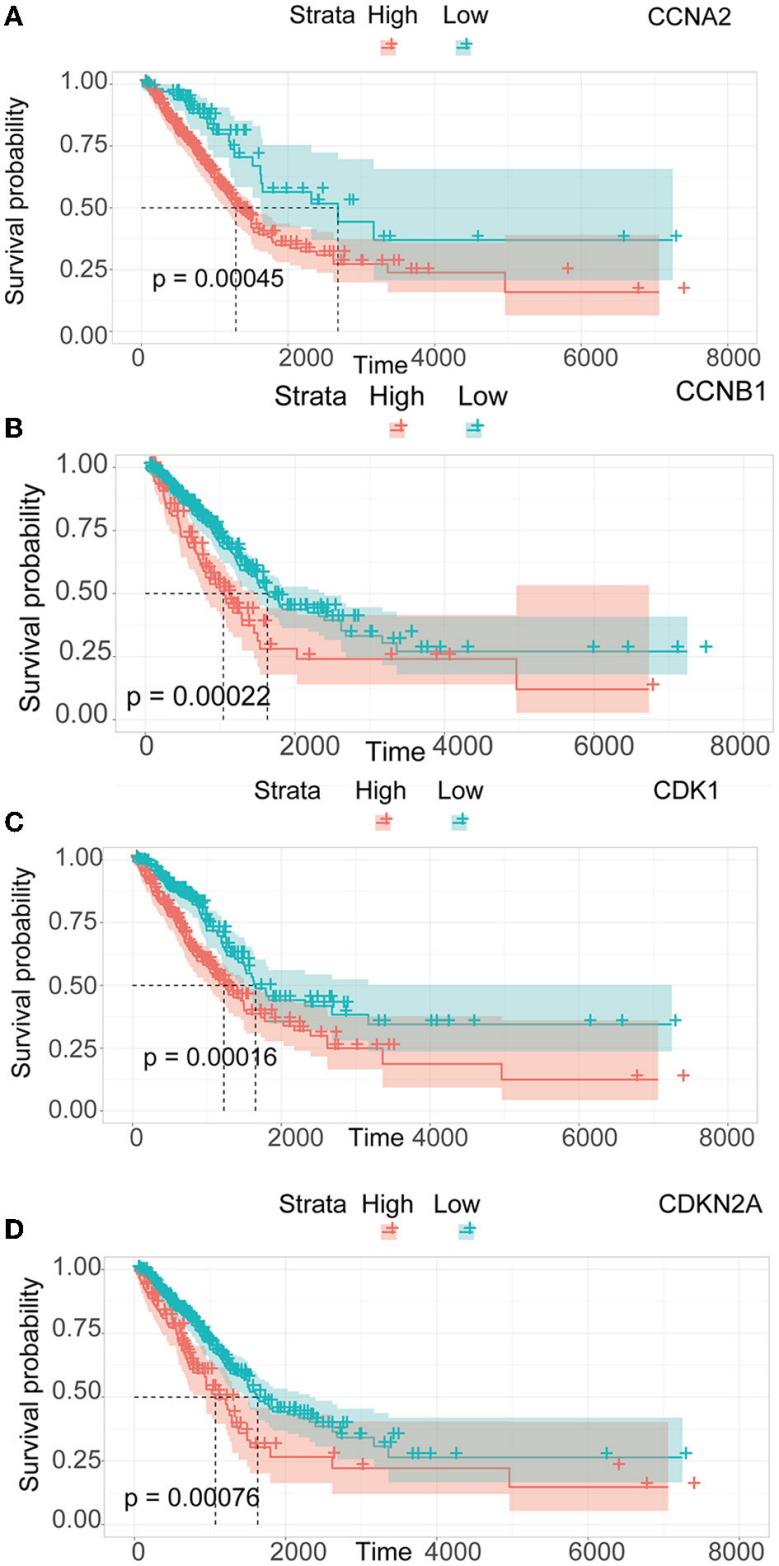
Survival analysis of LUAD. **(A)** Survival analysis of *CCNA2*. **(B)** Survival analysis of *CCNB1*. **(C)** Survival analysis of *CDK1*. **(D)** Survival analysis of *CDKN2A*.

**Figure 8:**
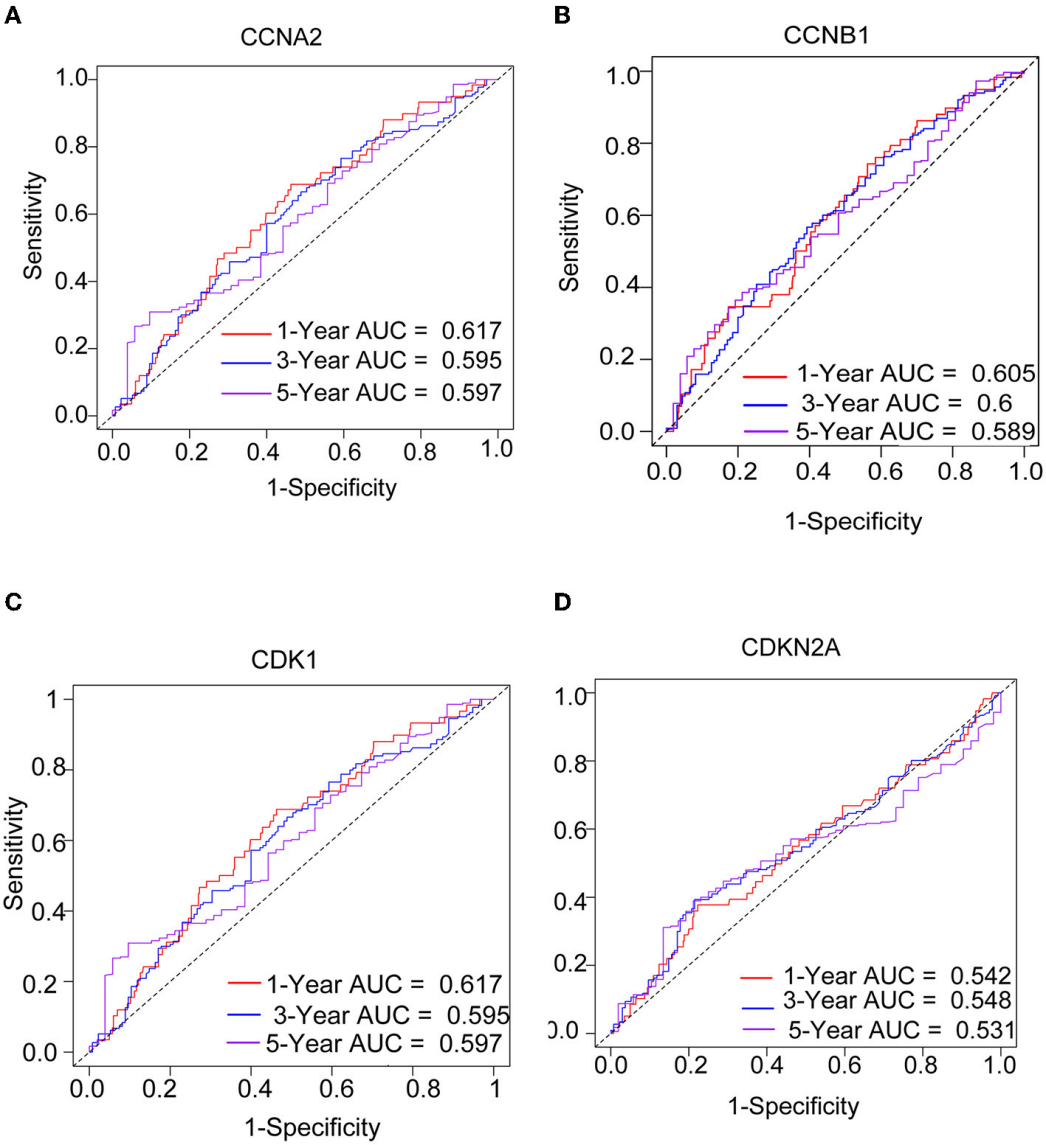
The ROC curves of four hub genes for predicting 1, 3, and 5-year mortality risk in the TCGA-LUAD dataset. **(A)** *CCNA2*. **(B)** *CCNB1*. **(C)** *CDK1*. **(D)** *CDKN2A*.

We performed a survival study where patients were classified into high-risk and low-risk groups depending on their gene expression level to confirm the prognosis of the four hub genes in LUAD. The findings revealed that hub genes with high expression have a poor prognosis in LUAD (Figure 7). It was revealed that the OS time of patients with a high risk score was significantly shorter than that of patients with a low risk score. The AUC values of 1,3 and 5 years of four hub genes were about 0.5–0.6 (Figure 8). By plotting KM curves and univariate Cox regression analysis, we obtained 5-year AUC, HR and 95% CI (Supplementary Table 1).

### 3.5. Immune infiltration analysis in LUAD and AIS

**Figure 9:**
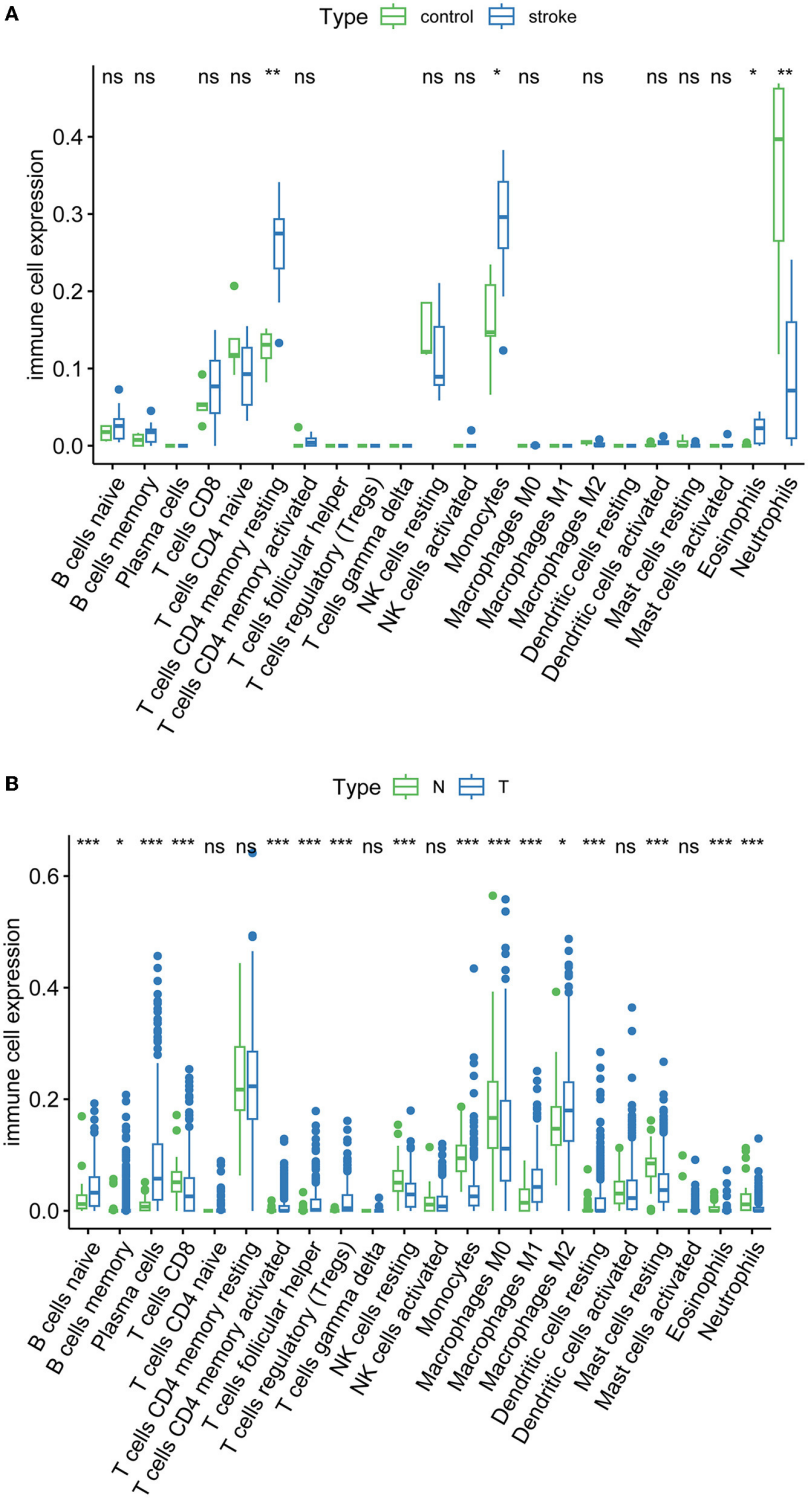
Immune infiltration of AIS and LUAD. **(A)** AIS. **(B)** LUAD (*, *P* < 0.05; **, *P* < 0.01; ***, *P* < 0.001; ns, not significant; N, normal; T, tumor).

**Figure 10:**
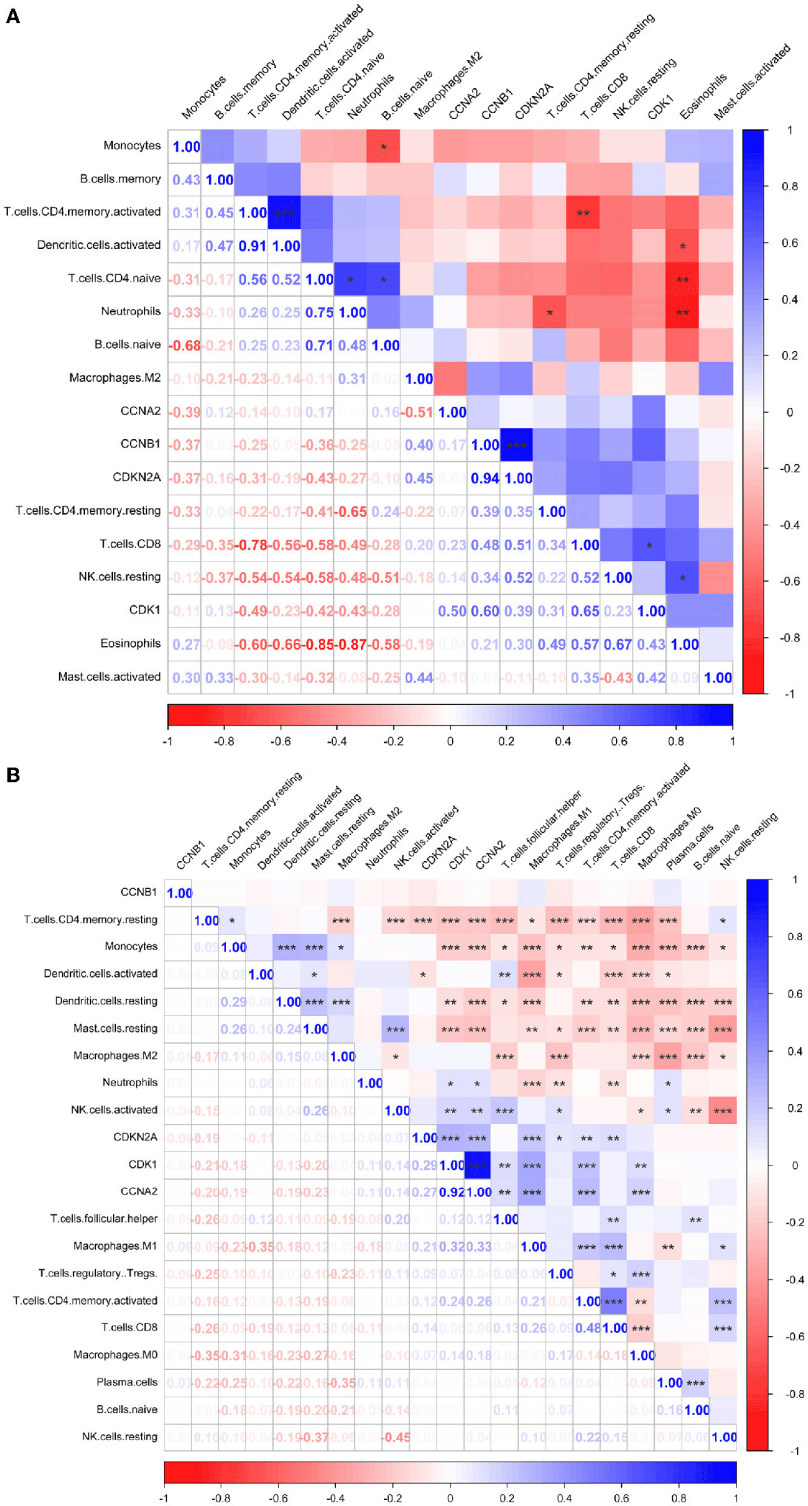
Immune correlation heatmaps of four hub genes in AIS and LUAD. **(A)** AIS. **(B)** LUAD (*, *P* < 0.05; **, *P* < 0.01; ***, *P* < 0.001).

Immune infiltration is essential for the onset, prognosis, and treatment of numerous disorders. While the control group had higher levels of neutrophils, AIS patients had higher levels of resident memory CD4^+^ T cells, monocytes, and eosinophils (Figure 9A). As seen in the image, several immune cells are involved in the formation of LUAD (Figure 9B). In AIS patients, only *CDK1* is associated with CD8^+^ T cells (Figure 10A). In LUAD patients, infiltration levels of resident memory CD4^+^ T cells, and activated dendritic cells were positively linked with *CDKN2A* overexpression. The immunological infiltration of resident memory CD4^+^ T cells, macrophage, activated dendritic cells, mast cells resting, activated NK cells and neutrophils were favorably connected with *CDK1* and *CCNA2* overexpression. However, it was not discovered that *CCNB1* was connected to immune cells (Figure 10B).

### 3.6. Molecular docking of curcumin with hub genes

**Figure 11:**
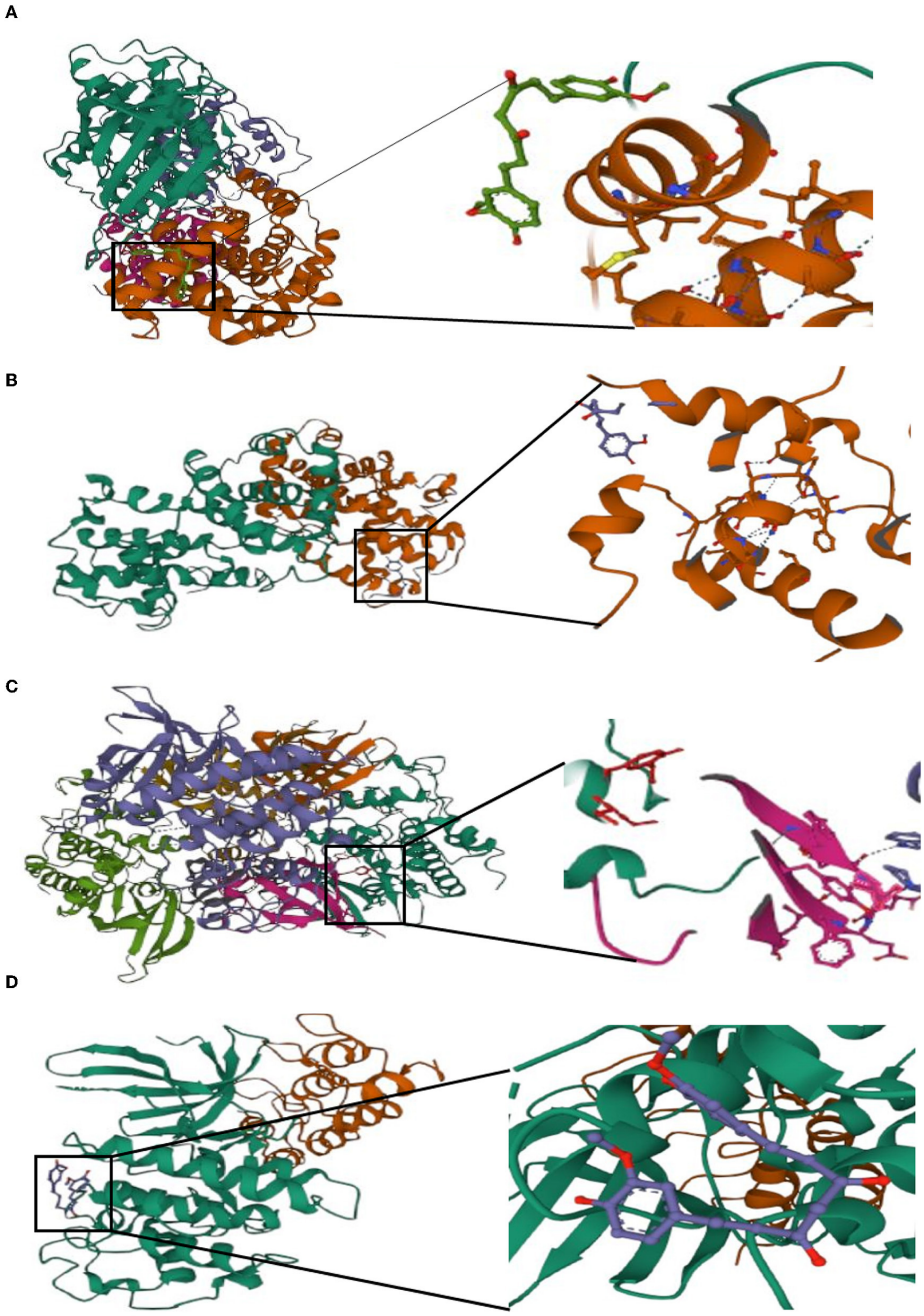
Molecular docking of curcumin with four hub genes. **(A)** *CCNA2*. **(B)** *CCNB1*. **(C)** *CDK1*. **(D)** *CDKN2A*.

Potential medicines were identified based on transcriptome features in the DSigDB database of Enrichr. Curcumin is considered as a potential drug for AIS treatment and later analysis. Moreover, we predicted the binding mechanism of curcumin with four hub genes using molecular docking. It is commonly accepted that the likelihood of action increases with decreasing ligand and receptor binding energies. The binding energies of curcumin with four hub genes (*CCNA2, CCNB1, CDK1, CDKN2A*) were −6.999 kcal/mol, −5.87 kcal/mol, −7.1 kcal/mol, −5.366 kcal/ mol, respectively. These findings demonstrate the highly stable binding of curcumin to four hub genes (Figure 11).

### 3.7. DEGs of different etiologies of AIS

According to the analysis above, *CCNA2, CCNB1, CDK1, CDKN2A* are the hub genes for AIS and LUAD. Results of DEGs of AIS caused by atherosclerosis and cardiac embolism shown by heatmaps (Supplementary Figure 5).

## 4. Discussion

The leading cause of adult disability and the second leading cause of death globally, behind ischemic heart disease, is stroke (24). Nearly 85% of lung cancer cases are non-small cell lung cancer (NSCLC), with the most prevalent histological subtype, LUAD, having a high mortality and recurrence rate (25). Stroke patients have an increased incidence of cancer, including lung cancer (26). A previous clinical study indicated that AIS may be a unique precursor to LUAD. In patients with LUAD combined with AIS, LUAD occurs due to the occurrence of AIS. The prevalence of lung cancer in the stroke cohort was 5.3 per 1000 person-years (27, 28). Lung cancer patients are most likely to experience a stroke within one year after diagnosis. The risk of stroke occurring in lung cancer patients is more than two times higher than that in people without cancer (HR 2.40, 95% CI 1.53–3.78) (23, 29). However, the causal relationship, genetic mechanisms, and their interactions between LUAD and AIS are still unclear. It is crucial to study the biological processes that underlie AIS and LUAD. The cerebral atherosclerosis-related gene *PITX2, RGS7, NKX2-5, NKX2-5*, and *ZFHX3* are involved in AIS. The genes of AIS brought on by cardiogenic embolism are *TM4SF4-TM4SF1, EDNRA, HDAC9-TWIST1*, and *LINC01492* (30). Our study shows that the hub genes of AIS with LUAD are *CCNA2, CCNB1, CDK1*, and *CDKN2A*. Meanwhile, our findings suggest that LUAD-induced AIS is distinct from AIS produced by large artery atherosclerosis and cardiac embolism in terms of genes. These genes' prognostic and diagnostic capabilities in LUAD were examined, and their mRNA levels were significantly elevated in AIS and LUAD. Herein, we investigated the link between AIS and LUAD using our four hub genes and explored the underlying biological mechanisms shared between these two diseases.

GO, KEGG, and DO enrichment analyses were performed on the 372 DEGs that intersecting both datasets. DEGs are mainly enriched in nuclear division, organelle fission and mitotic nuclear division, and they are associated with the biological behavior of LUAD and the neuroprotective inhibition observed in AIS. The primary enriched pathways in KEGG are the cell cycle, cellular senescence, and the HIF-1 signaling pathway. The cellular senescence pathway was highlighted as the main contributor of this study, and the overexpression of four cellular senescence-related genes was closely related to AIS and LUAD. HIF-1 was found to have a positive connection with infarct size in AIS (31). However, HIF's impact on neuronal survival following a stroke is still debatable (32). HIF-1-α is associated with aggressiveness in lung cancer (33). Our investigation revealed that the DEGs were linked to many malignancies.

According to one definition, cellular senescence is an ongoing proliferative arrest brought on by various stressors. Furthermore, senescence and hyperplastic pathology are both related to a type of stress response known as cellular senescence (34). Numerous neurological diseases, including neurodegenerative conditions like Alzheimer's disease, Parkinson's disease, and stroke, are linked to cellular senescence (35). Cellular senescence of the cells happens as AIS, which develops and progresses (36). AIS and neurological damage are impacted by the senescence-associated secretory phenotype (SASP) (37). Cellular senescence is considered the new hallmark of cancer, as malignant and non-malignant tumor cells develop a SASP that stimulates cancer recurrence and metastasis (38). However, it was once believed that cellular senescence contributed to the prevention of tumors; therefore, the mechanism of cellular senescence in cancer is not welldefined and should be further explored (39). Recently, it was demonstrated that a high expression of cellular senescence genes correlates with a poor LUAD prognosis (40). This is in line with our study, where our four hub genes *CCNA2, CCNB1, CDKN2A*, and *CDK1* were related to cellular senescence pathways. We investigated genes that were highly expressed in LUAD and that were linked to poor prognosis. It is crucial to deepen our understanding of how cellular senescence in AIS and LUAD functions, how it interacts with the immune system, and how it affects prognosis because the mechanism underlying these diseases has not yet been fully uncovered.

*CCNB1* combines with *CDK1* to form a complex that enables cells to enter the G2/M phase to promote mitosis (41, 42). Numerous malignancies have high *CCNB1* expression (43), increase apoptosis and cell death via controlling the p53 signaling pathway (44), accumulate in the degenerating brain regions of stroke patients, and can participate in neuronal death (45). The regulation of the mitotic cell cycle is influenced by *CDK1* (46). According to studies, *CDK1* may contribute to stroke through an oxidative mode of damage (47) and may also be a potential biomarker for NSCLC (48). *CDK1* is upregulated in patients with LUAD and accelerates tumor progression (49). *CCNB1* and *CDK1* enable the sustained proliferation of NSCLC by regulating the pRb protein (50). In our study, survival analysis showed that LUAD and AIS patients with overexpression of *CCNA2, CCNB1*, and *CDK1* had a poor prognosis and that these genes can be used as predictors of prognosis. In addition, these genes have high diagnostic abilities in patients with LUAD and AIS and could later serve as biomarkers for predicting these two diseases.

The cell cycle is regulated by *CDKN2A*, a cyclin-dependent kinase inhibitor that encodes the p16 protein (51). *CDKN2A* is a ferroptosis and cuproptosis gene (52, 53). When iron-dependent lipid hydroperoxides build up to deadly amounts, controlled cell death known as ferroptosis occurs (54). Cuproptosis is another type in which excess copper leads to cell death by aggregation of mitochondrial proteins (55). According to reports, *CDKN2A* is a locus for AIS risk. It has been linked to an elevated risk of AIS in the Han Chinese population and in native West African men. Genetic variation at the *CDKN2A* locus also predicts stroke in hypertensive patients (56–58). Lung cancer is associated with genetic mutations in *CDKN2A*, such as genomic deletions (59). The high expression of *CDKN2A* in LUAD is correlated with a bad prognosis. High *CDKN2A* expression may be associated with increased immune cell numbers, immune checkpoint enhancement, and elevated chemokine levels (60). *CDKN2A* is often regarded as a tumor suppressor gene; however, hypermethylated *CDKN2A* may be responsible for poor cancer prognosis (61). These four hub genes were verified in the HPA database and discovered to be related to LUAD prognosis, indicating they are crucial to the onset and development of LUAD. Curcumin has been shown to have neuroprotective and neuroregenerative properties. It can also be utilized as a drug for the treatment of ischemic stroke (62). This is in line with our findings, which shows that curcumin has a strong affinity to four hub genes. Curcumin might be a potential therapeutic target for the therapy of AIS, according to our drug prediction and molecular docking results.

This research has several restrictions. First, this study only employed a few samples. Second, we did not confirm the hub genes discovered in this study based on data from earlier experiments. However, their expression levels were validated.

According to our research, *CCNA2, CCNB1, CDKN2A*, and *CDK1* are significant components in AIS and LUAD. With multiple analyses, we explored AIS and LUAD pathogenesis as well as their common molecular mechanisms and observed high expression of these four hub genes, high diagnostic power in patients, and poor prognosis. Our results shed new light on improving AIS and LUAD prognosis. Further research into early intervention and treatment using our identified hub genes is warranted. Overall, this study identified *CCNA2, CCNB1, CDKN2A*, and *CDK1* as hub genes involved in the cellular senescence pathway and may serve as diagnostic and prognostic indicators for AIS and LUAD.

## Data availability statement

The original contributions presented in the study are included in the article/Supplementary material, further inquiries can be directed to the corresponding author.

## Author contributions

LC and R-XQ conceived the study. R-XQ, YY, and J-FC contributed to the data analysis and interpretation of the results. L-JH, WX, Q-CQ, X-JL, X-YL, X-YH, and M-SX wrote the manuscript. LC, R-XQ, YY, J-FC, L-JH, WX, Q-CQ, X-JL, X-YL, X-YH, and M-SX contributed to its editing. All authors contributed to the article and approved the submitted version.
