# Supplementary material for: Transcriptomic analysis reveals the potential biological mechanism of AIS and lung adenocarcinoma
Source: Front Neurol. 2023 May 17;14:1119160. doi: 10.3389/fneur.2023.1119160 (PMC10229805; doi:10.3389/fneur.2023.1119160)
Supplement: Supplementary file 1 [file Data_Sheet_1.pdf]

Supplementary Material

Transcriptomic analysis reveals the potential biological mechanism of AIS and lung adenocarcinoma

Rong-Xing Qin*, Yue Yang, Jia-Feng Chen, Li-Juan Huang, Wei Xu, Qing-Chun Qin, Xiao-Jun Liang, Xin-Yu Lai, Xiao-Ying Huang, Min-Shan Xie, Li Chen*

*** Correspondence:** Li Chen, e-mail: chenliqfkk@163.com

1 Supplementary Figures and Tables

1.1 Supplementary Figures

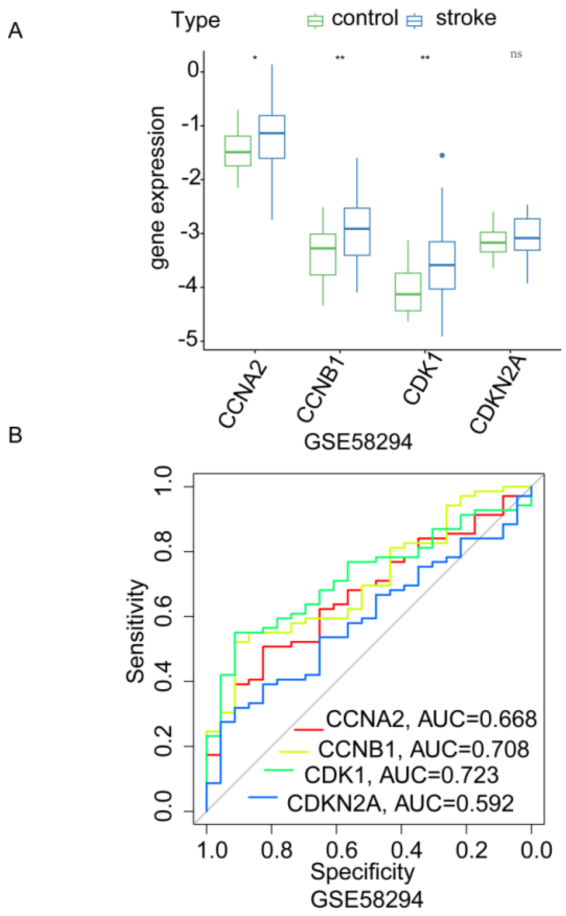

Supplementary Figure 1. Boxplots and ROC curves of four hub genes in AIS of GSE58294 datasets. (A). Boxplots of four hub genes in AIS. (B). ROC curves of four hub genes in AIS. (*, $P < 0.05$; **, $P < 0.01$; ns, not significant)

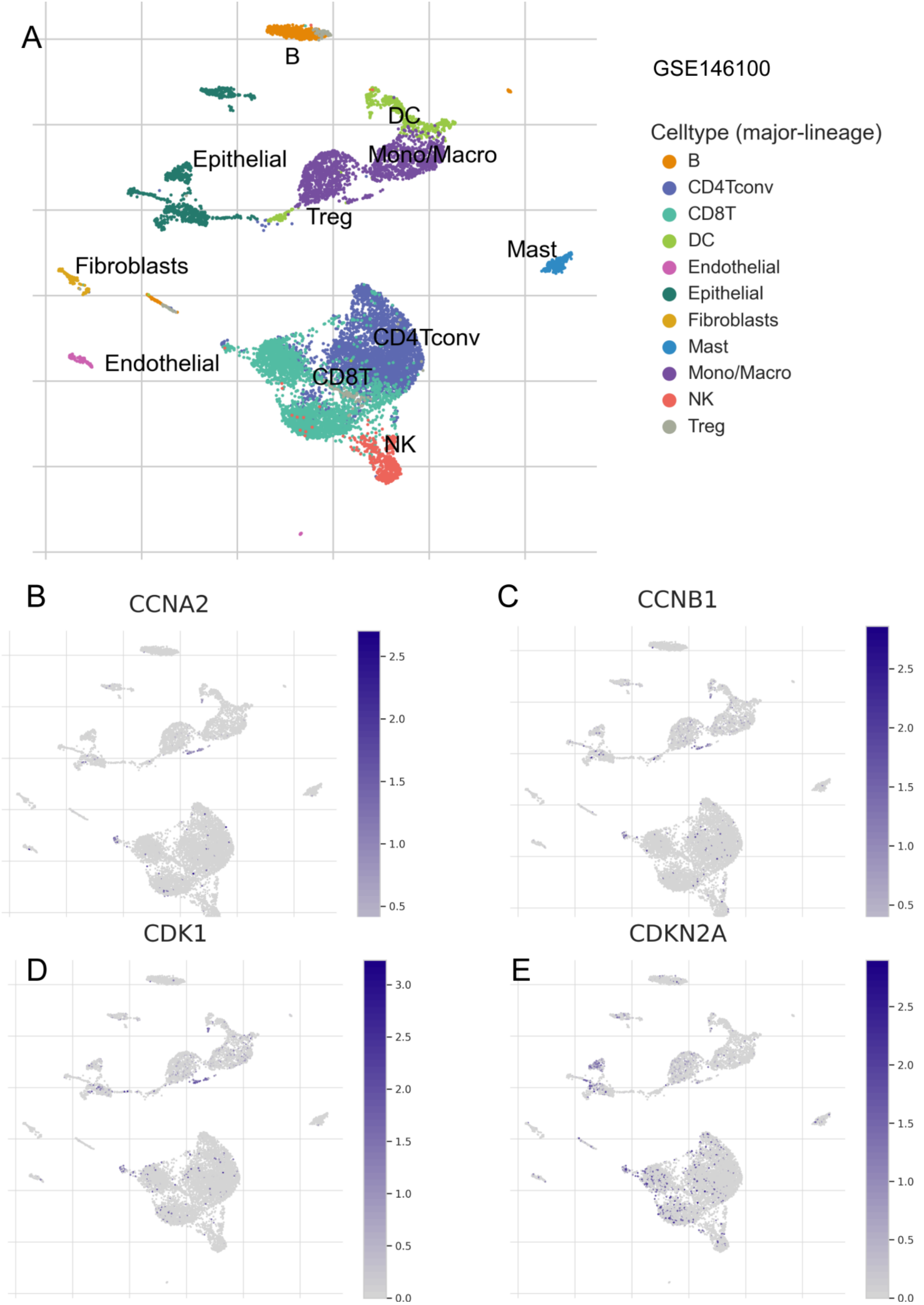

Supplementary Figure 2. The expression of genes in different cell types was analyzed using the single-cell dataset GSE146100. (A). Visualization of 11 clusters. (B). Distribution of *CCNA2*, *CCNB1*, *CDK1*, and *CDKN2A* in 11 clusters.

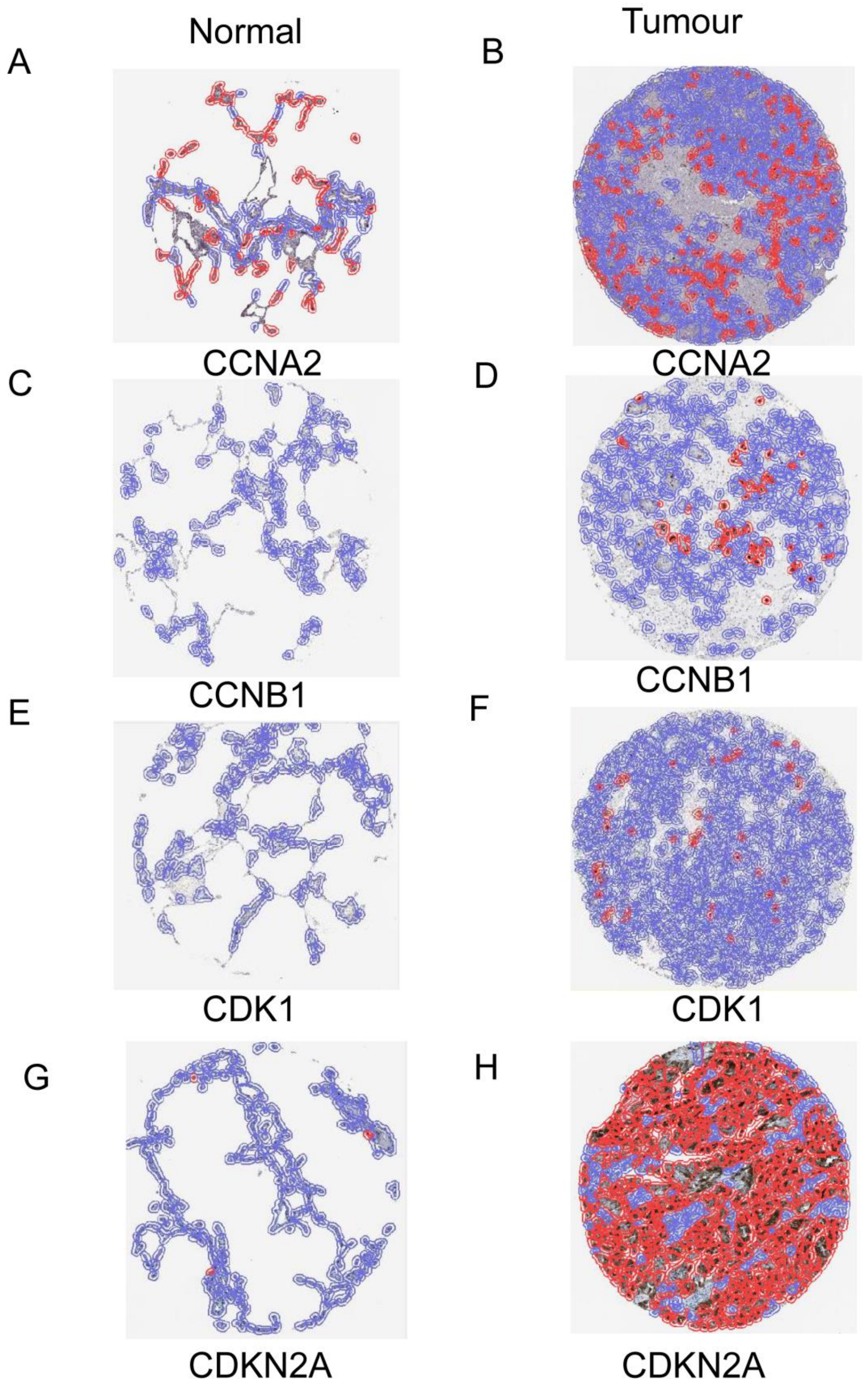

Supplementary Figure 3. The immunohistochemistry (IHC) image analysis findings were examined by the QuPath program. (A-H) was the positive cell recognition result of QuPath in LUAD and normal lung tissue. Red is positive cells, and blue is negative cells.

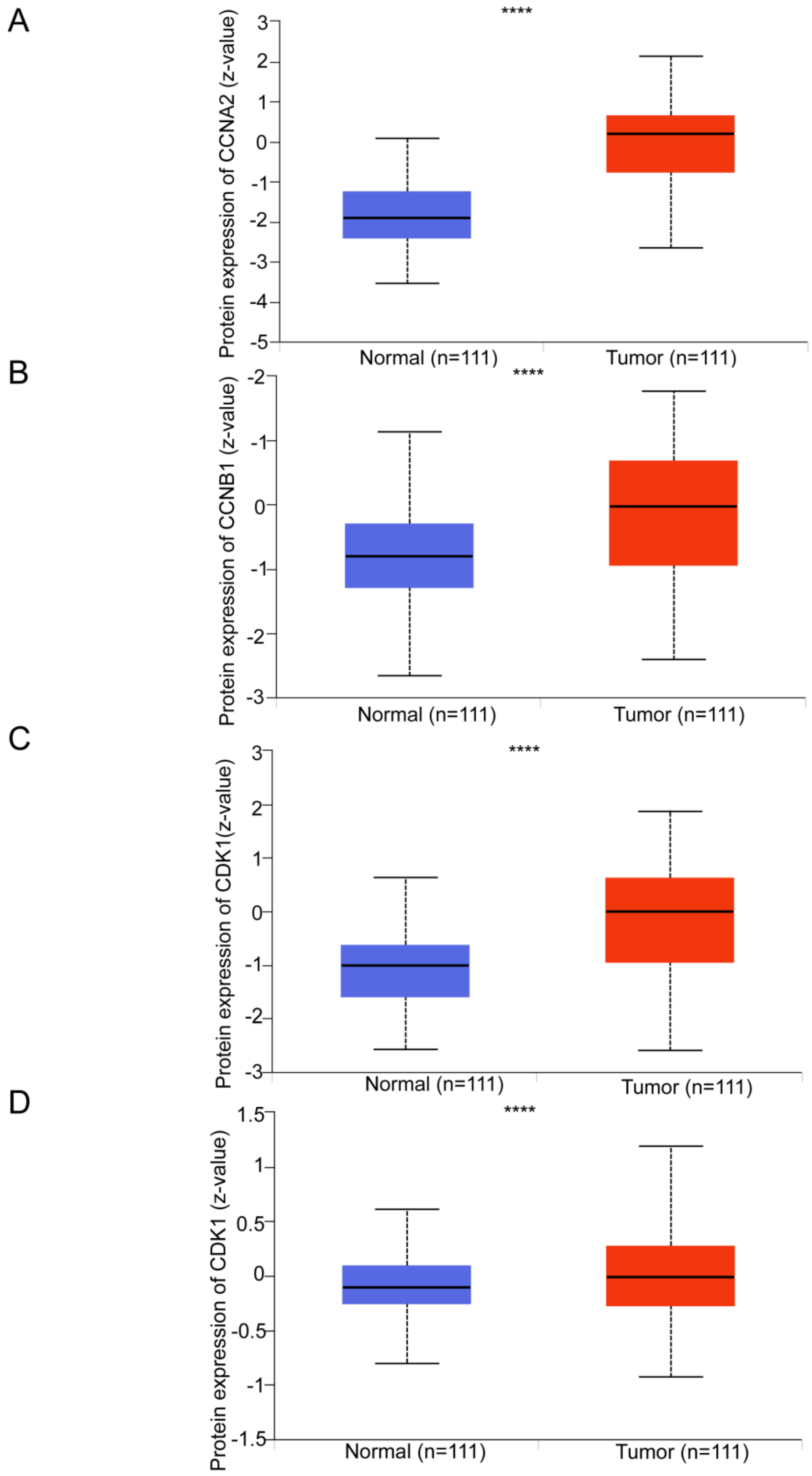

Supplementary Figure 4. The protein levels of *CCNA2*, *CCNB1*, *CDK1*, and *CDKN2A* in normal tissues and LUAD. (A) *CCNA2*, (B) *CCNB1*, (C) *CDK1*, (D) *CDKN2A*, ****, P < 0.0001.

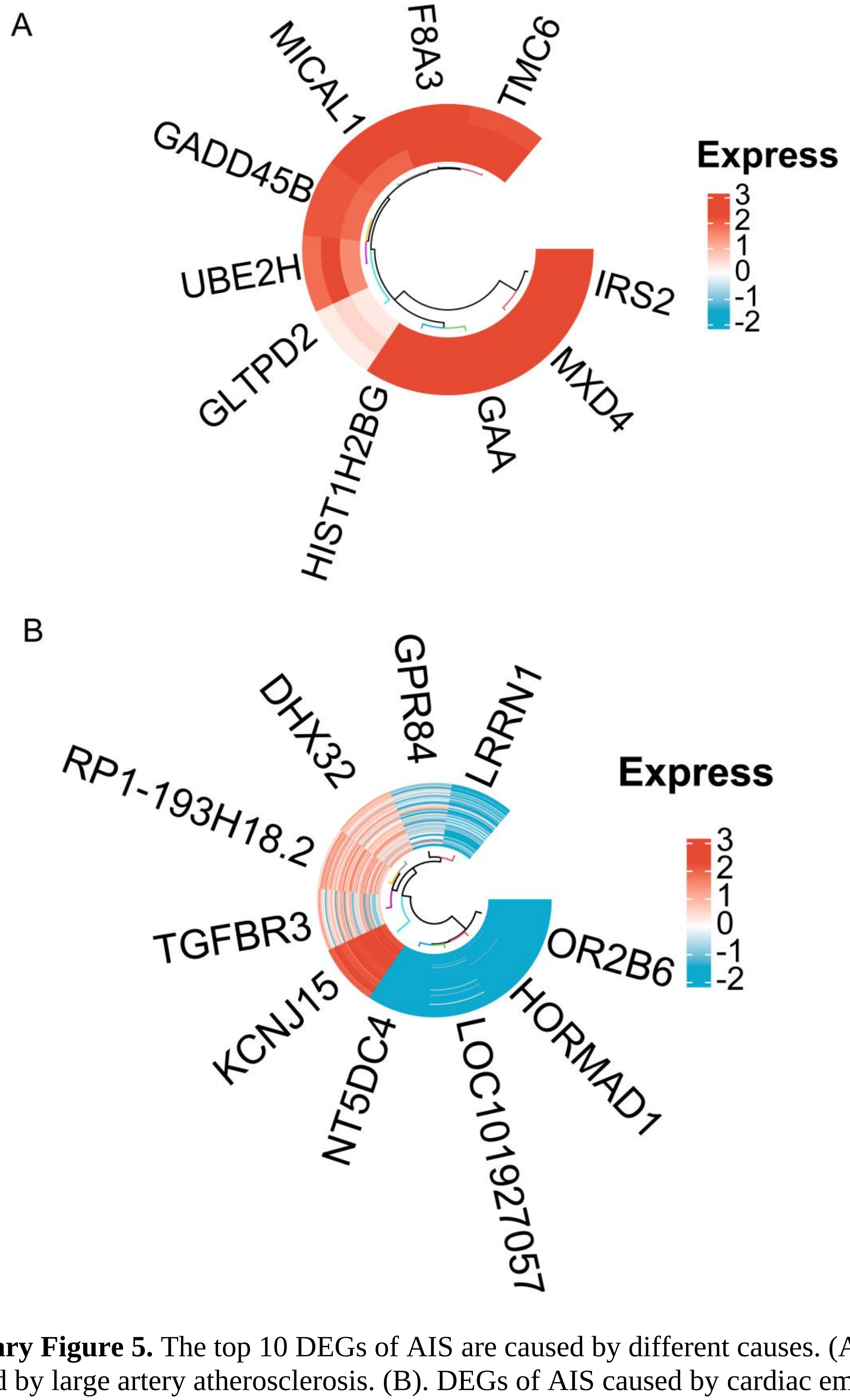

Supplementary Figure 5. The top 10 DEGs of AIS are caused by different causes. (A). DEGs of AIS caused by large artery atherosclerosis. (B). DEGs of AIS caused by cardiac embolism.

1.2 Supplementary Tables

Supplementary Table 1: Prognostic information of four hub genes in 5 validation datasets of LUAD. (Note: Some datasets have no *CDK1* gene, and NA indicates no result.)

ID	KM-pvalue	5-year-AUC	HR	95%CI	cox-pvalue
GSE42127-*CCNA2*	0.0029	0.608	1.27	1.028-1.581	0.026
GSE42127-*CCNB1*	0.00057	0.596	1.39	1.054-1.853	0.019
GSE42127-*CDKN2A*	0.0011	0.495	NA	NA	0.11
GSE68465-*CCNA2*	0.0057	0.603	2.53	1.467-4.368	0.0008
GSE68465-*CCNB1*	<0.0001	0.592	1.68	1.109-2.557	0.0143
GSE68465-*CDK1*	<0.0001	0.615	2.30	1.472-3.623	0.0002
GSE68465-*CDKN2A*	0.0044	0.502	NA	NA	0.26
GSE50081-*CCNA2*	<0.0001	0.976	1.36	1.072-1.735	0.0113
GSE50081-*CCNB1*	0.00042	0.967	1.42	1.123-1.811	0.0035
GSE50081-*CDK1*	0.0058	0.967	1.34	1.006-1.798	0.044
GSE50081-*CDKN2A*	0.0059	0.919	NA	NA	0.32
GSE13231-*CCNA2*	<0.0001	0.685	1.58	1.242-2.016	0.0002
GSE13231-*CCNB1*	0.00064	0.673	1.81	1.353-2.44	<0.0001
GSE13231-*CDK1*	0.00091	0.666	1.57	1.222-2.034	0.0004
GSE13231-*CDKN2A*	0.044	0.507	NA	NA	0.72
GSE31210-*CCNA2*	<0.0001	0.68	4.48	1.319-15.234	0.01
GSE31210-*CCNB1*	<0.0001	0.698	4.44	1.712-11.539	0.002
GSE31210-*CDK1*	0.00012	0.706	4.99	1.915-12.998	0.0009
GSE31210-*CDKN2A*	0.084	0.479	NA	NA	0.88
